# Investigation of radiomic signatures for local recurrence using primary tumor texture analysis in oropharyngeal head and neck cancer patients

**DOI:** 10.1038/s41598-017-14687-0

**Published:** 2018-01-24

**Authors:** M. D. Anderson Cancer Center Head and Neck Quantitative Imaging Working Group, Hesham Elhalawani, Aasheesh Kanwar, Abdallah S. R. Mohamed, Aubrey White, James Zafereo, Andrew Wong, Joel Berends, Shady Abohashem, Bowman Williams, Jeremy M. Aymard, Subha Perni, Jay Messer, Ben Warren, Bassem Youssef, Pei Yang, Mohamed A. M. Meheissen, Mona Kamal, Baher Elgohari, Rachel B. Ger, Carlos E. Cardenas, Xenia Fave, Lifei Zhang, Dennis Mackin, G. Elisabeta Marai, David M. Vock, Guadalupe M. Canahuate, Stephen Y. Lai, G. Brandon Gunn, Adam S. Garden, David I. Rosenthal, Laurence Court, Clifton D. Fuller

**Affiliations:** 1 Department of Radiation Oncology, The University of Texas MD Anderson Cancer Center, Houston, TX USA; 2 Texas Tech University Health Sciences Center School of Medicine, Lubbock, TX USA; 3 Department of Clinical Oncology, University of Alexandria, Alexandria, EG Egypt; 4 McGovern Medical School at University of Texas Health Science Center at Houston (UTHealth), Houston, TX USA; 5 University of Texas Health Science Center, San Antonio, TX USA; 6 Department of Cardiology, Harvard Medical School and Massachusetts General Hospital, Boston, MA USA; 7 Furman University, Greenville, SC USA; 8 Abilene Christian University, Abilene, TX USA; 9 Columbia College of Physicians and Surgeons, New York, MA USA; 10 Department of Radiation Oncology, American University of Beirut, Beirut, Lebanon; 11 Hunan Cancer Hospital, the affiliated cancer hospital of Xiangya School of Medicine, Central South University, Changsha, China; 12 Department of Clinical Oncology and Nuclear Medicine, Ain Shams University, Cairo, Egypt; 13 Clinical Oncology and Nuclear Medicine Department, University of Mansoura, Mansoura, Egypt; 14 Department of Radiation Physics, University of Texas MD Anderson Cancer Center, Houston, TX USA; 15 Graduate School of Biomedical Sciences, Houston, Texas USA; 16 Department of Computer Science, University of Illinois at Chicago, Chicago, Illinois USA; 17 Division of Biostatistics, University of Minnesota School of Public Health, Minneapolis, Minnesota USA; 18 Department of Electrical & Computer Engineering, University of Iowa, Iowa City, IA USA; 19 Department of Head and Neck Surgery, The University of Texas MD Anderson Cancer Center, Houston, TX USA; 20 Medical Physics Program, The University of Texas Graduate School of Biomedical Sciences, Houston, TX USA

## Abstract

Radiomics is one such “big data” approach that applies advanced image refining/data characterization algorithms to generate imaging features that can quantitatively classify tumor phenotypes in a non-invasive manner. We hypothesize that certain textural features of oropharyngeal cancer (OPC) primary tumors will have statistically significant correlations to patient outcomes such as local control. Patients from an IRB-approved database dispositioned to (chemo)radiotherapy for locally advanced OPC were included in this retrospective series. Pretreatment contrast CT scans were extracted and radiomics-based analysis of gross tumor volume of the primary disease (GTVp) were performed using imaging biomarker explorer (IBEX) software that runs in Matlab platform. Data set was randomly divided into a training dataset and test and tuning holdback dataset. Machine learning methods were applied to yield a radiomic signature consisting of features with minimal overlap and maximum prognostic significance. The radiomic signature was adapted to discriminate patients, in concordance with other key clinical prognosticators. 465 patients were available for analysis. A signature composed of 2 radiomic features from pre-therapy imaging was derived, based on the Intensity Direct and Neighbor Intensity Difference methods. Analysis of resultant groupings showed robust discrimination of recurrence probability and Kaplan-Meier-estimated local control rate (LCR) differences between “favorable” and “unfavorable” clusters were noted.

## Introduction

Recent investigations into radiomics, which is the extraction of quantitative imaging features from existing computed tomography (CT), magnetic resonance imaging (MRI) or positron-emission tomography (PET) images, has resulted in the capacity to discriminate potentially meaningful phenotypic differences in imaging which are not readily apparent to the human eye^1,2^. Radiomics holds substantive promise in that it allows the capacity to leverage existing technologies (such as CT acquired for diagnostic or planning purposes) for prognostic or predictive evaluation of patient outcomes^3^.

Currently, while large-scale data sets and machine learning techniques have been used to identify potential imaging signatures related to patient outcomes, the direct mechanistic underpinning of these radiomics-based image features has not been ascertained^4^. Preclinical investigations using animal models have shown that proliferative features and hypoxia are likely to be contributors to radiomic signatures of interest, and represent the vast majority of data presented^3,5^. More recent leaps in the domain of radiomics use the overall survival of patient populations as an endpoint and apply radiomics analysis to primary tumor volumes^6,7^.

Towards this end, we sought to investigate whether by limiting outcome information to specific events in the primary tumor volume, and attempting to correlate these site-specific local failures with discrete imaging features within gross tumor volumes, we might potentially observe radiomics-based image features, notwithstanding the complexities of composite endpoints. That is to say, identification of imaging features in the primary tumor volume which are correlated with survival obfuscates whether the pattern of failure is dependent on local recurrence and progression, regional /nodal failure/progression, or distant metastasis. Similarly, use of survival endpoints obscures whether salvage therapy successfully mediates the potential mortality inducing effects of a recurrence or second primary event. Consequently, we sought to isolate local features through our standardized extraction process, which might be directly linked to local phenomena in the same spatial region.

The specific aims of the current study therefore included:

1. Determine whether radiomics features of the primary tumor can predict for local control in OPC patients treated with definitive chemo/intensity-modulated radiotherapy (IMRT).
2. Identify and validate composite radiomics-based image signature with potential predictive utility of local disease control.
3. Hypothesis generation for future prospective studies.

## Results

### Patients

Four-hundred sixty-five patients were included for analysis in the exploratory dataset. The whole dataset was divided into 3 cohorts, including: training portion (255 patients), a second tuning set (165 patients) as well as a smaller test set (45 patients). The cohort had a median age of 58 and included predominantly males (86%) who had smoked at least once (58.9%) with more than half exhibiting base of tongue cancer (53.3%), at an advanced stage “AJCC stage III-IV” (96.2%) for which chemoradiation was prescribed (89%), either: sequentially (11.2%), concurrently (53.8%) or both (24%). Human papilloma virus (HPV) status was retrieved for only 62% of the cohort, being positive in more than half the cohort. According to our institutional policy, the presence of HPV DNA is tested by use of the *in situ* hybridization (ISH)–catalyzed signal amplification method for biotinylated probes and/or the expression status of p16 via immunohistochemistry (IHC). Detailed demographics, disease and treatment characteristics are further depicted for the 3 cohorts combined and individually in Table 1.

**Table 1:** Demographics, disease and treatment characteristics for the three subsets.

Characteristics	Training set n (%)	Tuning set n (%)	Test set n (%)	All 3 sets combined n (%)
**Sex**				
*M*	229 (89.8)	131 (79.4)	40 (88.9)	400 (86)
*F*	26 (10.2)	34 (20.6)	5 (11.1)	65 (14)
Age at diagnosis, years: median (range)	57 (28–83)	59.2 (29.5–88.5)	58 (43–89)	58 (28–89)
**Ethnicity**				
*White*	240 (94.1)	145 (87.9)	42 (93.3)	427 (91.8)
*Black*	5^38^	9 (5.5)	2 (4.5)	16 (3.4)
*Hispanic*	9 (3.6)	8 (4.8)	1 (2.2)	18 (4)
*Native American*	1 (0.3)	0 (0)	0 (0)	1 (0.2)
*Asian*	0 (0)	3 (1.8)	0 (0)	3 (0.6)
**Smoking status**				
*Former*	83 (32.5)	67 (40.6)	19 (42.2)	169 (36.3)
*Current*	54 (21.2)	40 (24.2)	11 (24.5)	105 (22.6)
*Never*	118 (46.3)	58 (35.2)	15 (33.3)	191 (41.1)
**Tumor side**				
*Right*	122 (47.8)	85 (51.5)	25 (55.6)	232 (49.9)
*Left*	123 (48.2)	79 (47.9)	19 (42.2)	221 (47.5)
*Central*	8 (3.1)	0 (0)	0 (0)	8 (1.7)
*Bilateral*	2 (0.8)	1 (0.6)	0 (0)	3 (0.7)
*More than one distinct contralateral subsites*	0 (0)	0 (0)	1 (2.2)	1 (0.2)
**Subsite within the oropharynx**				
*Base of tongue*	141 (55.3)	83 (50.3)	24 (53.3)	248 (53.3)
*Tonsil*	106 (41.6)	59 (35.8)	17 (37.9)	182 (39.1)
*Soft palate*	3 (1.2)	3 (1.8)	2 (4.4)	8 (1.7)
*Lateral oropharyngeal wall*	3 (1.2)	0 (0)	1 (2.2)	4 (0.9)
*Posterior oropharyngeal wall*	2 (0.8)	0 (0)	0 (0)	2 (0.4)
*Glossopharyngeal sulcus*	0 (0)	8 (4.8)	0 (0)	8 (1.7)
*Vallecula*	0 (0)	2 (1.2)	0 (0)	2 (0.4)
*More than one distinct ipsi/contralateral subsites*	0 (0)	10 (6.1)	1 (2.2)	11 (2.5)
**T category**				
*T1*	36 (14.2)	53 (32.1)	3 (6.7)	92 (19.8)
*T2*	114 (44.7)	65 (39.4)	21 (46.7)	200 (43)
*T3*	67 (26.3)	28 (17)	11 (24.4)	106 (22.8)
*T4*	38 (14.9)	19 (11.5)	10 (22.2)	67 (14.4)
**N category**				
*N0*	15 (5.9)	20 (12.1)	5 (11.1)	40 (8.6)
*N1*	13 (5.1)	28 (17)	5 (11.1)	46 (9.9)
*N2*	220 (86.3)	113 (68.5)	31 (69)	364 (78.3)
*N3*	7 (2.7)	4 (2.4)	2 (4.4)	13 (2.8)
Nx	0 (0)	0 (0)	2 (4.4)	2 (0.4)
**AJCC Stage**				
*I*	0 (0)	3 (1.8)	0 (0)	3 (0.6)
*II*	4 (1.6)	8 (4.8)	3 (6.7)	15 (3.2)
*III*	21 (8.2)	35 (21.2)	6 (13.3)	62 (13.3)
*IV*	230 (90.2)	119 (72.1)	56 (80)	385 (82.9)
**HPV status**				
*Positive*	103 (40.3)	128 (77.6)	6 (13.3)	237 (51)
*Negative*	11 (4.3)	37 (22.4)	3 (6.7)	51 (11)
*Unknown*	141 (55.3)	0 (0)	36 (80)	177 (38)
**Therapeutic combinations**				
*Radiation alone*	2 (0.8)	40 (24.2)	9 (20)	51 (11)
*Induction chemotherapy* (*IC*) *then radiation alone*	45 (17.6)	4 (2.4)	3 (6.7)	52 (11.2)
*Concurrent chemoradiotherapy* (*CCRT*)	127 (49.8)	96 (58.2)	27 (60)	250 (53.8)
*IC then CCRT*	81 (31.8)	25 (15.2)	6 (13.3)	12 (24)
**Neck dissection after IMRT**				
*Yes*	65 (25.5)	27 (16.4)	8 (17.8)	100 (21.5)
*No*	190 (74.5)	138 (83.6)	37 (82.2)	365 (78.5)
Radiation dose^39^ (median)	70 Gy	70 Gy	70	70 Gy
Radiation fractions (median)	33	33	33	33
**Vital status**				
*Alive*	226 (88.6)	136 (82.4)	29 (64.4)	391 (84.1)
*Deceased*	29 (11.4)	29 (17.6)	16 (35.6)	74 (15.9)
**Local control**				
*Yes*	237 (92.9)	154 (93.3)	41 (91.1)	432 (92.9)
*No*	18 (7.1)	11 (6.7)	4 (8.9)	33 (7.1)
Time to local failure, median (range)	56 (1–101)	50.9 (1.8–137)	58 (1–117)	54.9 (1–137)

### Radiomics-based image feature identification

The primary tumor gross tumor volume (GTVp) was contoured and analyzed using the IBEX software from Zhang *et al*.^8^. For each GTVp, 134 features were extracted. These features can be separated into 4 domains: intensity direct, intensity histogram, shape and texture. For the intensity domain, 42 features were calculated based on the direct intensity values and from a histogram of intensity values for each region of interest^9^. For the shape domain, 18 features were calculated based on the 3-dimensional rendering of each ROI. For the texture domain, several different methods of texture analysis were applied, to obtain the remaining 74 feature descriptors. These include the gray-level co-occurrence matrix (GLCM) in 3 dimensions and 2.5 dimensions (where features are calculated on each 2-D slice and averaged across the z-axis of all slices included in the ROI), the neighborhood intensity different matrix (NIDM) in 2.5 dimensions and 3 dimensions, and gray-level run length matrix (GLRLM) in 2.5 dimensions. With respect to the filter-based domain, laplacian of Gaussian (LoG) and butterworth filters were exclusively applied to the ROI before features from the aforementioned domains other than shape were calculated, along with voxel size resampling was unanimously applied before all features analysis.

Applying the aforementioned preprocessing filters can yield more than 11,000 permutated versions of the original 134 features for each patient’s primary tumor. So given the low event rate and to evade over-training, we ran Bootstrap resampled recursive partitioning analysis (RPA) and regression models yielded a group of candidate quantitative imaging features when applied to the training set and a second tuning set. This was based on a martingale residual that was generated from clinical prognosticator-based multivariate Cox model. Of the 465 patients, 33 (7.1%) had locally recurrent disease, distributed as follows: 11 (6.7%) in the training set, 4 (8.9%) in the tuning set and 33 (7.1%) in the test set. RPA identified radiomics-based classifiers, predictive of local control, derived from GTVp.

**Figure 1:**
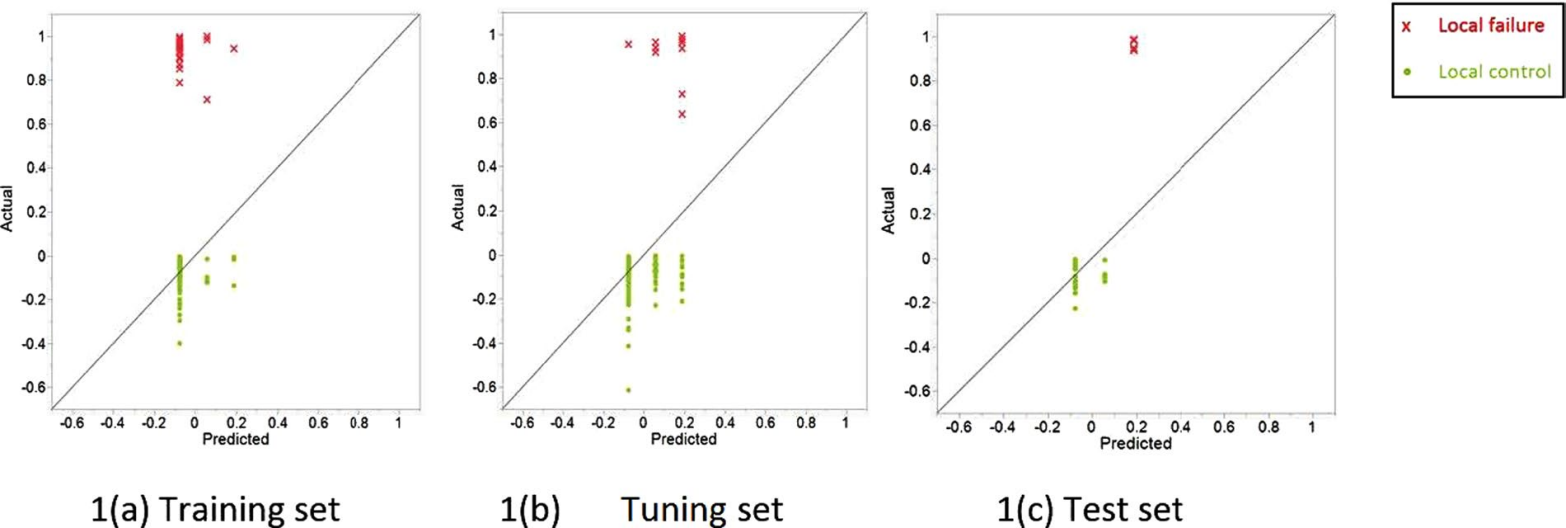
‘Actual Martingale residual’ by ‘predicted value from random forest’ plot of time-to-local failure for the three sets based on the radiomic signature.

Further modelling and pruning the decision tree yielded a two-feature profile that could be correlated to local recurrence after definitive (chemo)IMRT in our dataset. The two features are: Intensity Direct Local Range Max (LRM) and Neighbor Intensity Difference 2.5 Complexity with cut-off values corresponding to 1616 and 457808 respectively. After training a multivariable Cox proportional hazards model on the training cohort, the model was verified on both the ‘test’ and ‘tuning’ sets. The predictive performance of the model was consistent among the three subsets. Figure 1 graphically demonstrates actual martingale residuals of local control for the 3 subsets of patients plotted against those predicted according to the proposed radiomic signature. This actual by predicted plot is a capable statistical tool for visualization of goodness of fit of decision tree for continuous responses.

Furthermore, survival curves were plotted for the three cohorts testing the discriminating power of the two radiomics-based image features, individually or combined in a semi-factorial design. The combined radiomic classifier composed of (i) **Intensity Direct LRM < 1616 and NID 2**.**5 Complexity < 457808** persistently revealed discriminatory results for prediction of more favorable local tumor control across the three groups, versus other individual features or other combinations. The other two classifiers were: (ii) combined radiomic signature composed of **Intensity Direct LRM < 1616 and NID 2**.**5 Complexity ≥ 457808** and (iii) **Intensity Direct LRM > 1616**.

**Figure 2:**
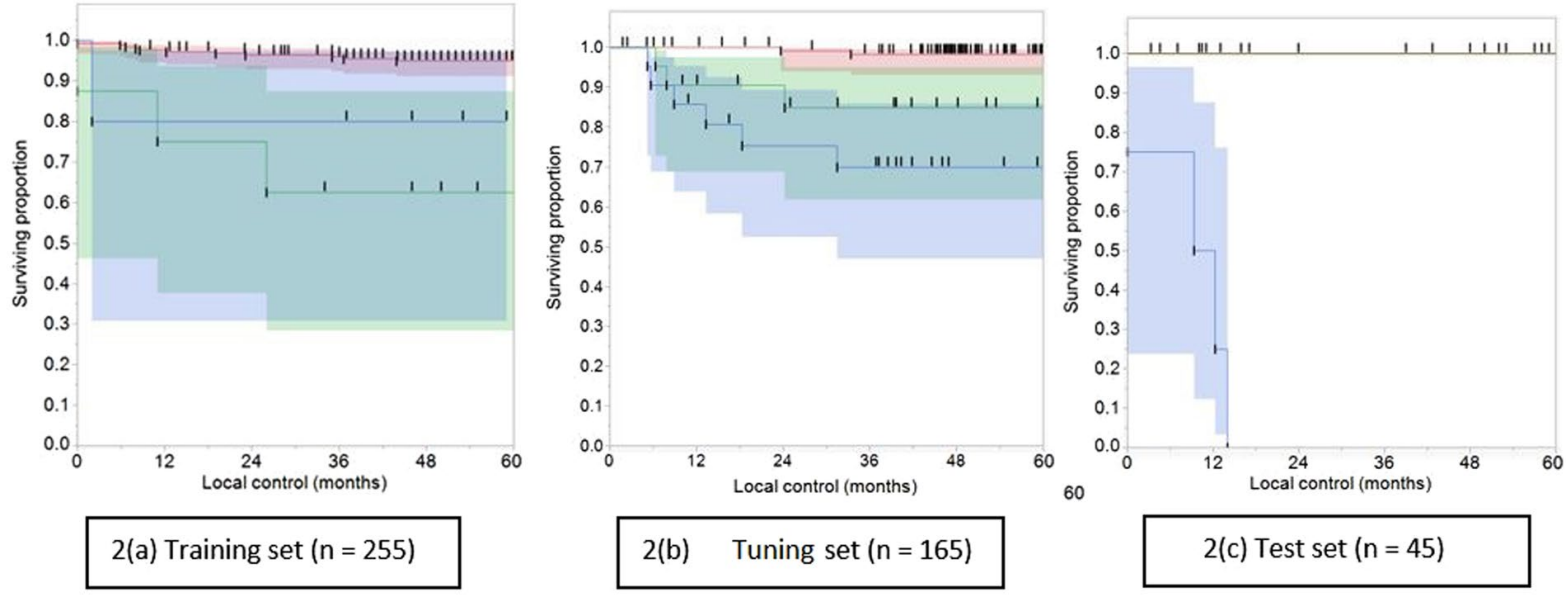
Local tumor control by permutations of radiomics-based image features combination. Kaplan-Meier curve showing local control (in months) for patients classified against different values. (Intensity Direct LRM = Intensity Direct Local Range Max, NID2.5 Complexity = Neighbor Intensity Difference 2.5 Complexity) We give the number of subjects at 0, 12, 24, 36, 48 and 60 months follow-up who were still at risk (i.e., not censored or been diagnosed with local failure or died). **2**(**a**) **Training set** (**n = 255**). **2**(**b**) **Tuning set** (**n = 165**). **2**(**c**) **Test set** (**n = 45**) (Note that the two upper lines in this curve are overlapping given the absence of events, i.e. local failure in this permutation).

The discriminatory value was very striking in the training set where the 5-year local control rate (LCR) in the subset of patients grouped according to the radiomic classifier (i) was 94%, surpassing the 5-year LCR for classifier (ii) and (iii) patients being 62% and 80%, respectively as shown in Fig. 2(a). This difference in 5-year LCR was statistically significant by both Log-Rank and Wilcoxon tests, with p < 0.001 in both tests. Likewise, the tuning set patients classified in the same fashion showed peculiar 5-year LCRs of 98%, 86% and 70%, respectively, again with statistically significant Log-Rank and Wilcoxon tests (p < 0.001), as shown in Fig. 2(b). Noteworthy, the ranking of the risk groups changed from training to tuning set from (i), (iii), (ii) to (i), (ii), (iii).

The test set showed similar trend, but no conclusions can be drawn from this, given the low rate of events which were all encountered in group (iii), which can be explained by the small number of this set (n = 45). However, this was also depicted in Fig. 2(c).

Moreover, in the pursuit of developing a clinical/imaging nomogram predictive of local IMRT outcomes in locally advanced H&N cancer patients, known H&N cancer clinical prognosticators (as defined per ‘Methods’) were integrated along with the derived radiomic classifiers in a robust statistical analysis. The ultimate goal was to identify a constellation of potential clinical and imaging biomarkers of IMRT response, investigating their predictive performance individually or as aggregates, taking into consideration False Discovery Rate (FDR). Towards that end, Effect Likelihood Ratio tests and Wald tests were applied to these biomarkers using a full-factorial design, where all possible combinations of these biomarkers were tested against each other^10^. Akaike information criterion (AIC) and Bayesian information criterion (BIC) were our benchmark for model selection among the proposed set of models by the aforementioned statistical tests, with the lowest AIC and BIC being preferred.

Our radiomic classifier was named “Combination radiomic signature” and was tested against and in combination with the clinical prognosticators in an iterative manner. Wald and effect likelihood ratio tests were applied to find out if radiomic signature and/or clinical variables have a true added value on the model-based stratification of patients in terms of local control. Again these parametric statistical tests were run on ‘training’ and ‘tuning’ sets; excluding the ‘test’ set given the smaller portion of patients with known HPV status. Across all iterations, the radiomic classifier persistently showed the highest statistical significance (p = 0.001), as compared to other statistically significant clinical factors, including HPV status (p = 0.042), Age (p = 0.035) according to Wald test. Similarly, effect likelihood ratio test revealed that our radiomic classifier, HPV status, age, smoking status and therapeutic combination were statistically significant with corresponding p values of 0.0001, 0.026, 0.026, 0.026 and 0.039, respectively, as shown in Tables 2 and 3, respectively.

**Table 2:** Wald Test (DF = degree of freedom).

Source	Number of parameters	DF	Wald ChiSquare	Prob > ChiSq
Combination radiomic signature	2	2	13.187	0.001*
HPV Status	2	2	6.337	0.042*
Smoking status	2	2	5.820	0.055
Age	1	1	4.379	0.036*
Sex	1	1	0.193	0.661
Therapeutic Combination	3	3	2.910	0.406
T	3	3	0.165	0.983
N	4	4	0.592	0.964

**Table 3:** Effect Likelihood Ratio Tests (L-R = Likelihood ratio).

Source	Number of parameters	DF	L-R ChiSquare	Prob > ChiSq
Combination radiomic signature	2	2	17.826	0.0001*
HPV Status	2	2	7.334	0.026*
Smoking status	2	2	7.289	0.026*
Age	1	1	4.973	0.026*
Sex	1	1	0.191	0.662
Therapeutic Combination	3	3	8.363	0.039*
T	3	3	0.166	0.983
N	4	4	2.5801	0.630

Moreover, our radiomic classifier was the most predictive of time-to-local recurrence even more predictive than established clinical risk factors with the lowest AIC and BIC values across different iterations. This also corresponds to the lowest, most statistically significant FDR p-value as shown in Table 4.

**Table 4:** Effect summary in the light of False Discovery Rate (FDR) test.

Source	FDR LogWorth		FDR P Value
Combination radiomic signature	3.312		0.0004
Smoking status	1.334		0.046
Age	1.011		0.098
Therapeutic Combination	0.985		0.103

## Discussion

Medical imaging is regarded as a cornerstone in the algorithm of management and subsequent response evaluation of cancers^11^. For years, tumor size was almost the only quantitative standard that was extracted from different imaging modalities, and changes over the course of treatment were the guide to treatment planning in the population-based cancer management fashion^12^.

In the era of personalized cancer medicine, innovative sources of meaningful data are critically needed. Radiomics is one such “big data” approach that applies advanced image refining/data characterization algorithms to generate imaging features that may be used to quantitatively classify tumor phenotypes in a noninvasive manner^13^. We hypothesize that certain local textural features of primary tumors will have statistically significant correlations to patient outcomes such as local control.

Head and neck cancers represent a significant amount of cancers in the United States with an estimated 50,000 new cases every year and rising incidence according to the projections into the future, especially the oropharyngeal cancers, given its parallel to the climbing incidence of HPV-16 genotype infections^14^. This correlation called up for more efforts to adapt the conventional AJCC/UICC staging to accommodate the HPV risk variable^15^.

Similarly, quantitative imaging features are tested in this study to develop unique signatures to further gratify risk stratification and subsequent management planning. Radiomics has the potential to individualize patient treatment by using images that are already being routinely acquired. Imaging, contrary to tissue biopsy, is capable of capturing the entire tumor volume and reflecting the intra-tumor heterogeneity. Our current work pivots around mining CT images looking for quantifiable imaging biomarkers of potential clinical use. CT imaging was opted because, besides its wide availability and ease of use, it is an indispensable imaging modality in the OPC staging work-up algorithm, either alone or as a part of PET/CT examinations^16^.

To the best of our knowledge, this project is one of the leading undertakings in the assessment of local treatment outcomes of OPC, based on locally-derived radiomics-based image features of contrast-enhanced CT; in terms of local control, for future personalized radiation therapy applications. A signature composed of two radiomics-based image features from pre-IMRT CT imaging was an independent predictor of local control. These texture parameters were derived based on statistical approaches, like absolute gradient, which measures the spatial variation of grey-level values across the image, and co-occurrence matrix, which depends on the use of second-order statistics of the gray-scale image histograms. The former method yielded “Intensity Direct LRM”, which is defined by the IBEX software user manual as the median among all voxels’ range value per voxel in its neighborhood region^17^. Whereas the latter generated “NID 2.5 Complexity”, which refers to the visual information content of a texture^18^.

This methodological study showcases the usefulness of decision trees in oropharynx tumor control modeling after (chemo)radiotherapy. Moreover, this radiomic signature demonstrates the possible imperceptible association between invisible tumor phenotypic characteristics, as captured by radiomics analysis, and tumor biologic features controlling subsequent response to radiotherapy.

Our work provides preliminary conclusions that, after external validation and curation, will pave the road for more radiomic classifiers studies with subsequent clinical utility as one of the determinants in the radiation therapy planning and dose prescription decision algorithm in a prospective fashion. Similar classifiers can potentially serve as ahead of time alert that a subset of OPC patients are at higher risker of post-IMRT local recurrence if treated using the current generalized dose prescription and planning guidelines. The conceivable impact of such radiomics classifiers, derived from inherently ‘local’ phenotypic tumor characteristics, can mimic the impact of tumor biology on OPC treatment process. Trials investigating IMRT dose intensification in HPV-negative OPC is one example to mention^19^.

Traditionally, machine-learning methods in the radiomics domain have been heavily invested in exploration of prognostic imaging biomarkers for H&N cancers. The efforts by Aerts *et al*. were one of the earliest mature advents in the field of prognostic radiomics. They identified a set of radiomic features, representing the chief feature categories that were proved to be linked to oncologic outcomes in independent sets of lung and H&N cancer patients^18^. Along the same lines, Parmar and his colleagues pinpointed 3 radiomics-based image feature selection approaches that showed great proficiency and consistency for prediction of 3-year OS in H&N cancers^20^.

Also, we have to highlight some of the possible caveats in our study and how we managed to overcome them or alternatively point out the way to do so in future projects. First, this study may be limited by its retrospective design. Albeit, 3 cohorts were used for deep learning, training, tuning and test subsets, which accounted for a total of 465 OPC patients. Patient selection was carried out very meticulously to make up for lack of external validation set. Besides, further validation of this radiomics-based profile is planned on an external set of OPC patients with disease and treatment characteristics that are matched to our cohort’s.

Still, selecting a non-redundant assortment of radiomics-based image features from the very broad redundant set of features was very challenging. With such large complexity, the pitfall of over-fitting and subsequent “curse of dimensionality”, i.e. more features than samples may lead to weaker feature signal, was highly anticipated given previous trials^21^. We tried to overcome this by prioritizing the features in a systematic fashion applying machine learning techniques, using training, tuning and test sets.

Our approach relied on supervised techniques; building a model for rare events, i.e. local recurrence in OPC patients in the IMRT era, based on labeled data in a training set. Moreover, a second tuning set was used to estimate prediction error and tweak the feature selection for model optimization. Ultimately, a test set was used for assessing the strength and utility of a predictive relationship. These machine learning methods were thus employed to reduce the redundancy and maximize the statistical relevance of features. However, the radiomic signature IDLRM>1616 was associated with fairly inconsistent results, i.e. an intermediate outcome in the ‘training’ set and the worst outcome in both ‘tuning’ and ‘test’ sets. This can be explained -in part- by the relatively smaller size of the test set in addition to the non-identical make-up of the three sets; being curated from different institutional cohorts.

Moreover, the predictive capacity of the proposed multi-feature radiomic signature was tested in ‘training’ and ‘tuning’ sets against other well-established clinical prognosticators of the disease. These include: age, gender, HPV status, smoking status, T-category, N-category and AJCC stage. Again the ‘test’ set wasn’t included in this analysis given the smaller portion of patients with known HPV status. This might have predisposed to over-training of the model on ‘training’ and ‘tuning’ sets with subsequent overfittingThis radiomic signature added to the predictive capacity of the aforementioned prognostic factors combined. In our opinion, this provides a framework for a predictive clinical/imaging model that can be built up to a comprehensive nomogram by integrating other determinants, most importantly genetic and pathologic elements, among others. Not to mention the relative congruency of correlating ‘local’ radiomics-based image features to ‘local’ treatment outcome.

Specific to oropharynx radiomics, biology (HPV/p16) is such a strong driver in disease outcomes that imaging features should be always interpreted in congruence with corresponding HPV status^22^. In our cohort, HPV status was retrievable for 62% of the patients. For more consistent and representative analysis, when assessing the performance of our combined radiomic signature against HPV-based models, patients with unknown HPV status were censored. Otherwise, all patients were integrated into model building and comparison. Accordingly, we limited the comparison to ‘training’ and ‘tuning’ sets given the low proportion of subjects with known HPV status in the ‘test’ set. Further work comparing the relative performance of this radiomic signature to HPV status is needed.

Yet, there are possible uncertainties introduced from incongruent acquisition parameters or from the inherent variability between different vendors, or even between different models from the same vendor. Mackin *et al*. have shown that inter-scanner variability for some features can be greater than interpatient variability for those features in an NSCLC cohort^23^. The second preprocessing filter applied was the Weiner smoothing filter, which has been shown to decrease the range of variability in features between different CT manufacturers^24^. This highlights the importance of standardization of acquisition parameters and, if possible, machinery in future prospective studies.

Another potential source of uncertainty is the presence of digital artifacts due to the presence of dental hardware. Primary GTVs were contoured on image slices that don’t show visible metal artifacts, because these areas of markedly increased or decreased attenuation would have an effect on calculations based on HU intensity and gray level texture analysis. However, this might have a detrimental effect on shape calculations. We postulate that more studies should delve into studying the stability of radiomics-based image features calculated before and after applying Metal Artifacts Reduction Software, or ‘MARS’, to raw CT data^25^.

As discussed in the methods section, two filters were also applied as a preprocessing step before features were calculated. The first of these was the voxel size resampling feature to create uniform voxel sizes across the dataset. Previous work by *Fave et al*. have shown that resampling voxel sizes increases the reproducibility of features in test-retest datasets such as RIDER dataset^26^. In one iteration, image sets containing artifacts were excluded but all voxel sizes were allowed. In another iteration, image sets with voxel dimensions not equal to the standard of 0.048 cm × 0.048 cm were processed using a trilinear interpolation algorithm to resize voxels to be on identical size across the cohort.

Nonetheless, several variations in acquisition parameters in CT scans beyond manufacturer/model still exist. This is to be expected in a retrospectively acquired dataset. These include but not limited to peak tube voltage, tube current and acquisition mode whether axial or helical, which studies showed to play a role in substantial variations in radiomics-based image features for the same patient^27^.

The heterogeneity of imaging data might represent the most challenging limitation to the generalization of our prognostic model, although it is less profound than in the TCIA dataset. However, in a way, this pitfall can also be a strength in the findings in terms of its simulation to real clinical practice situation. Moreover, different radiomics software may handle calculations slightly differently. While the equations that are used are published (for example, in appendix of the Aerts Nature article and the feature references for IBEX), one has to delve into the actual matlab code and be able to understand it to actually determine whether the implementation of the calculations is actually equal/congruent. This is possible with open-source solutions such as IBEX and CERR but is limited in many other publications because they are not available to public^28^. We’ve used an open-source IBEX software that is freely available to the public so that others may evaluate the implementation of our radiomic profile, if they opt to.

This research work is a step towards filling the gap in large scale highly curated imaging/clinical data sets that allow further model development, risk stratification refinement, and/or scalable validation with sufficient statistical robustness. Radiomics is a “big data” approach to medical imaging that extracts a large amount of quantitative information from routine acquisitions for biomarker investigation. Incorporating medical imaging into the “big data” paradigm allows the expression of microscopic genomic and proteomics patterns in terms of macroscopic image-based features. Because the radiomics analysis is performed on images that are routinely acquired in clinical practice, it presents a cost-effective and highly feasible addition for clinical decision support.

In conclusion, this study demonstrates an association between locally-derived tumor radiomic features and local disease control for patients with locally advanced OPC after (chemo)radiotherapy. Moreover, similar radiomic signatures, if validated on various OPC cohorts of diverse biologic make-up, can be synergistically integrated along with known clinical prognisticators into a multi-factorial decision making tool towards personalized radiotherapy planning and delivery.

## Methods and Analysis

### Patients

Patients were retrieved from an internal University of Texas MD Anderson Cancer Center database after getting approved by the University of Texas MD Anderson Cancer Center Institutional review board (IRB). All methods for this study were performed in accordance with the University of Texas MD Anderson Cancer Center IRB guidelines and regulations. Being an HIPAA-compliant retrospective study waived the prerequisite for informed consent. All patients had biopsy-proven squamous cell carcinoma of the oropharynx who have been dispositioned to radiation therapy as a single or multimodality definitive therapy were included. We used the ICD 10 codes as a reference, when defining the anatomic subsites of the oropharynx, as follows: base of tongue (BOT), tonsil, soft palate, pharyngeal wall (posterior and/or lateral), glossopharyngeal sulcus (GPS), vallecula or other; in case subsites of origin were overriding, which is referred to in the ICD 10 coding system as “malignant neoplasm of overlapping sites of oropharynx”. All the eligible patients were treated using IMRT, with the exclusion of other radiotherapy techniques and modalities, like particle therapy.

**Figure 3:**
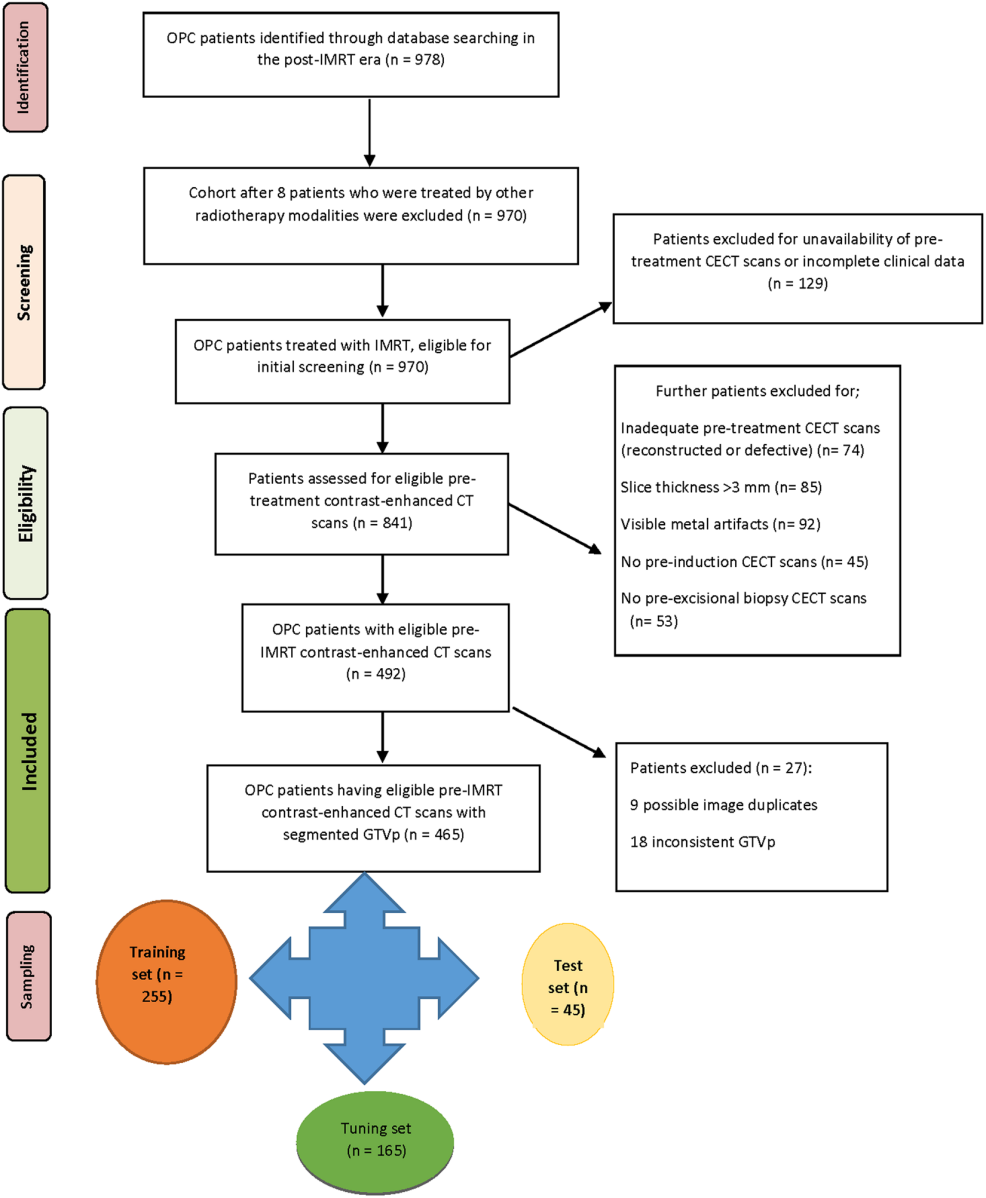
Flowchart of patient selection for inclusion.

After conforming to these inclusion criteria, the study data set of 465 patients with histopathologically-proven OPC was concluded. The CONSORT diagram representing the inclusion process of the study population is depicted in Fig. 3.

The whole dataset was divided into 3 cohorts, including: ‘training set’ (255 patients), a second ‘tuning set’ (165 patients), as well as a smaller ‘test set’ (45 patients).

### Endpoint

Each OPC patient was closely followed-up at regular intervals every 2–3 months during the first 2 years, then every 3–6 months during Years 3–5 post-treatment. Locoregional imaging studies (contrast-enhanced CT with or without MRI or PET-CT scans) were requested on a regular basis and/or if clinically required. Median follow-up duration for this cohort was 55 months.

Each treatment failure harbored at the primary (local) oropharyngeal site had to be proven pathologically (biopsy and/or resection) and/or radiologically to be counted in as a ‘local failure’. Otherwise, the patient was recognized as ‘locally controlled’^29^.

### CT Imaging Protocol

Contrast-enhanced CT images were performed independently in the course of pre-treatment diagnostic work-up according to institutional protocol. All CT scans, performed at our institution, were acquired with a multi-detector row CT scanner (Lightspeed 16, GE Healthcare, Milkwaukee, WI) with the following parameters: a 1–3 mm thick sections, with median section thickness of 1 mm, an X-ray tube current of 99–584 mA (median: 220 mA) at peak voltage 120–140 kVP. All images acquired at our institution were composed of 512 × 512 pixels and were acquired following a 90 second delay after intravenous contrast administration. 120cc of contrast was injected at a rate of 3cc/sec. To minimize variability caused by incongruent acquisition parameters, image resampling was performed based on voxel size and slice thickness parameters. Sixty-two patients had CT scans with a slice thickness that was not equal to 1 mm (range 0.5 mm to 3 mm). Likewise, 62 patients had CT scans with pixel dimensions that were not equal to 0.0488 cm × 0.0488 cm along the x- and y- axes respectively (range 0.0357 cm × 0.0357 cm – 0.0638 cm × 0.0638 cm) and a trilinear interpolation voxel resampling filter was applied to these studies using IBEX to yield voxel sizes congruent with the mode of the dataset.

### Image Segmentation

Individual pre-treatment contrast-enhanced CT imaging sets of the head and neck were evaluated beforehand, and the primary tumor lesions were spotted independently by two expert radiation oncologists, who were blinded to relevant clinical meta-data. Gross tumor volume (GTV) contours of the primary disease (GTVp) constituted our regions of interest (ROIs). Discrepancies were resolved by consensus or the call of a third expert radiation oncologist. Manual segmentation was performed using commercial treatment planning software VelocityAI™ 3.0.1 software (powered by VelocityGrid). Gross tumor volumes were segmented according to the ICRU 62/83 definition of “the gross demonstrable extent and location of the tumor”^30^. Findings from physical examination, endoscopy and other imaging modalities, like magnetic resonance imaging (MRI) and PET also guided the segmentation as per our previous projects^31^. In case tumor was partly obscured by visible metal artifacts, GTVp wasn’t segmented in the slices which included the defect. Computed tomography scans along with the curated ROIs were then extracted in digital imaging and communications and medicine (DICOM-RT) format.

### Radiomics analysis

Radiomics analysis was performed using the open-source institutionally-developed software “Imaging Biomarker Explorer” (IBEX), which utilizes the Matlab platform (Mathworks Inc, Natick, VA). The software (IBEX) is a freely available radiomics analysis platform developed by Zhang *et al*. at University of Texas MD Anderson Cancer Center. Computed tomography images in DICOM format were imported into IBEX. Likewise, GTVp contours in DICOM-RTSTRUCT format were imported into IBEX. Our analysis entailed the exploration of a group of agnostic imaging features that largely encompass intensity, shape and texture. These features are typically categorized into first, second and higher order texture features, based on the method applied to estimate how the pixels are inter-related^32^. First order features are solely based on intensity (HU) values and the shape of the ROI. These features are extracted either directly or from a histogram analysis prior to any mathematical transformation and regardless of spatial configuration. Intensity-based features such as entropy and variance address the overall dispersion of grey levels but given their nature, are limited regarding precise spatial distribution of gray levels within the tumor.

To quantify intratumoral heterogeneity incorporating spatial information, textural analysis was applied which constitutes the second-order statistical output. These entail approaches like gray level co-occurrence matrix (GLCM), gray level run length matrix (GLRLM), as well as neighbor intensity difference^32^. In these methods, a set of mathematical transformations was implemented to the images to develop a “parent matrix”. Using this parent matrix, a myriad of equations for features such as energy, entropy, dissimilarity, and correlation may be applied^33^. Voxel size was resampled in the three dimensions into constant values beforehand, via a trilinear interpolation preprocessing filter. Accordingly, voxel size was set to 0.488 mm in the x-dimension, 0.488 mm in the y-dimension and 1mm in the z-dimension as these were the modal values in the dataset.

Other preprocessing filters that were evaluated include a Laplacian of Gaussian filter and Butterworth smoothening filter^24,26^. With respect to the Laplacian of Gaussian (LoG) transformation, the filter was applied prior to calculation of intensity-based features. The standard deviation (sigma) of the LoG filter was manipulated from a size 0.5 voxels to 2.5 voxels, for a total of 5 iterations, based on previous work by Ganeshan *et al*.^34^.

For all intensity and texture features, an iteration using the Butterworth smoothing preprocessing filter was included to estimate the impact of smoothing or noise removal on radiomics-based image features. When calculating the Butterworth filters, the ROIs were padded to 512 × 512 pixels. With the cutoff frequency used in our analysis, the boundary effects were negligible. Moreover, the 2-dimensional butterworth filter was applied in conjugation with the 3-D voxel size resampling filter sequentially so that images were processed to uniform voxel sizes before the smoothing process. Many of the aforementioned algorithms had various parameters that can be modified to yield drastically different results. To that end, many of these features were exhaustively explored using multiple iterations of filters with varying parameters. Examples of parameters include neighborhood size (measured in voxels) or sigma, the size of the Gaussian filter as measured in millimeters.

A list of the evaluated image features is depicted in Supplementary Table (Table S1), chiefly based on the feature descriptions provided by the IBEX software developers. More elaborate definitions of these statistical texture features, along with relevant equations were also presented by Davnall *et al*.^35^.

### Statistical analysis

Bivariate plots of various quantitative imaging biomarkers (outputs of radiomics analysis algorithms) were dichotomized by the presence or absence of local disease recurrence at 5 years. Multivariate bootstrap resampled recursive partitioning analysis (RPA) was conducted to identify and test candidate quantitative imaging biomarkers associated with increased probability of local failure. Recursive partitioning analysis (also known as classification and regression trees) was selected over other parametric methodologies as it allows selection of candidate “thresholds” for continuous variables using a binary endpoint. Recursive partitioning analysis is especially robust when limited priors preclude knowledge-based selection of continuous candidate features, and is comparatively unaffected by multi-collinearity and/or potential hyper-dimensional interactions within/between candidate covariates. RPA and regression models were applied systematically in the following steps:

1. Candidate quantitative imaging features were explored using bootstrap resampled RPA for all given features for all patients, with the Martingale residual (“variability of time-to-local failure not explained by clinical factors”) generated from a multivariate Cox proportional hazards model as the discriminant variable^36,37^. The multivariate Cox model included the following conventional demographic and prognostic variables: sex, age, race, tumor subsite, T stage, N stage, AJCC stage, HPV status and smoking status.
2. A post-pruning approach was adopted where a minimum number of cases was imposed that once reached forces the termination of the RPA algorithm and hence features at upper nodes were selected as “best candidate features.
3. Integer thresholds for the discriminant variable (e.g. local control) within “best” candidate features using K-fold cross validation.
4. A predictive model for the discriminant variable (*i*.*e*. local control) was built by testing “best” candidate features and clinical variables on the training set. Stepwise nominal regression with Bayesian Information Criteria (BIC) minimization optimization were applied for model selection and comparison.
5. A second tuning set was used to estimate prediction error and tweak the feature selection for model optimization; the test set was ultimately used for assessing the strength and utility of a predictive relationship.
6. Population level estimates of local control probabilities were plotted using post hoc bootstrapped logistic probability models and subsequent unsupervised nonlinear curve fits similar to the methodology of Wedenberg.

For this exploratory analysis and model construction, uncorrected p-values are presented, with a priori p ≤ 0.05 considered for provisional statistical significance. Effect sizes, and LogWorth values (wherein LogWorth represents −log_10_[p-value], such that p = 0.01 is equivalent to a LogWorth of 2.0, p = 0.001 is denoted by LogWorth of 3.0, etc.) were applied.

The high dimensionality of the data produced in this analysis necessitated the implementation of advanced statistical methods to reduce the feature space to a meaningful, practical size. To that end, The feature space was consequently reduced to create a radiomics profile of the most statistically relevant features, for the patients to be stratified according to the ultimate radiomic signature. Local control as a direct outcome for radiation treatment was evaluated using Kaplan-Meier product limit curves using the radiomic signature for grouping. The prognostic performance of the derived radiomics model was then evaluated using Aikake Information criterion (AIC) for analysis. All statistical analysis was performed using commercial statistical analysis software (MatLab R2011a, Mathworks, Natick, MA; JMP Pro v12.1, SAS Institute, Cary, NC, USA; IBM SPSS 22.0, Chicago, IL) as well as *Stata*.

## Electronic supplementary material

Table S1. Computed tomography-derived intensity histogram, shape and texture analysis features set
