# Supplementary material for: Investigation of radiomic signatures for local recurrence using primary tumor texture analysis in oropharyngeal head and neck cancer patients
Source: Sci Rep. 2018 Jan 24;8:1524. doi: 10.1038/s41598-017-14687-0 (PMC5784146; doi:10.1038/s41598-017-14687-0)
Supplement: Supplementary file 1 — Table S1. Computed tomography-derived intensity histogram, shape and texture analysis features set [file 41598_2017_14687_MOESM1_ESM.pdf]

Title:

Investigation of radiomic signatures for local recurrence using primary tumor texture analysis in oropharyngeal head and neck cancer patients

Contributing authors:

Hesham Elhalawani, MD, MSc[1], Aasheesh Kanwar, BS[1,2], Abdallah S.R. Mohamed, MD, MSc[1,3], Aubrey White, BS[1,4], James Zafereo, BS[1,4], Andrew Wong, BS[1,5], Joel Berends, BS[1,5], Shady Abohashem, MD[1,6], Bowman Williams, BS[1,7], Jeremy M. Aymard, BS[1,8], Subha Perni, BS[1,9], Jay Messer[1], Ben Warren[1], Bassem Youssef[10], Pei Yang, MD, MPH[1,11], Mohamed Meheissen[1,3], Mona Kamal, MD, PhD[1,12] Baher Elgohari, MD, MSc[1,13], Rachel Ger, BS[14,15], Carlos Cardenas, BS[14], Xenia Fave, BS[14], Lifei Zhang, PhD[14], Dennis Mackin, PhD[14], G. Elisabeta Marai, PhD[16], David Vock, PhD[17], Guadalupe M. Canahuate, PhD[18], Stephen Y. Lai, MD, PhD[19], G. Brandon Gunn, MD[1], Adam S. Garden, MD[1], David I. Rosenthal, MD[1], Laurence Court, PhD[14], Clifton D. Fuller, MD, PhD[1,20*]

Affiliations:

1. Department of Radiation Oncology, The University of Texas MD Anderson Cancer Center, Houston, TX, USA.
2. Texas Tech University Health Sciences Center School of Medicine, Lubbock, TX, USA.
3. Department of Clinical Oncology, University of Alexandria, Alexandria, EG.
4. McGovern Medical School at University of Texas Health Science Center at Houston (UTHealth), Houston, TX, USA.
5. University of Texas Health Science Center, San Antonio, TX, USA.
6. Department of Cardiology, Harvard Medical School and Massachusetts General Hospital, Boston, MA, USA.
7. Furman University, Greenville, SC, USA.
8. Abilene Christian University, Abilene, TX, USA.
9. Columbia College of Physicians and Surgeons, New York, MA, USA.
10. Department of Radiation Oncology, American University of Beirut, Beirut, Lebanon.
11. Hunan Cancer Hospital, the affiliated cancer hospital of Xiangya School of Medicine, Central South University.
12. Department of Clinical Oncology and Nuclear Medicine, Ain Shams University, Cairo, Egypt.
13. Clinical Oncology and Nuclear Medicine Department, University of Mansoura, Mansoura, Egypt.
14. Department of Radiation Physics, University of Texas MD Anderson Cancer Center, Houston, TX, USA
15. Graduate School of Biomedical Sciences, Houston, Texas, USA

16. Department of Computer Science, University of Illinois at Chicago, Chicago, Illinois, USA.
17. Department of Biostatistics, University of Minnesota of Public Health, Minneapolis, Minnesota, USA.
18. Department of Electrical & Computer Engineering, University of Iowa, Iowa City, IA, USA.
19. Department of Head and Neck Surgery, The University of Texas MD Anderson Cancer Center, Houston, TX, USA.
20. Medical Physics Program, The University of Texas Graduate School of Biomedical Sciences, Houston, TX, USA.

***Corresponding authors**:

Correspondence to Clifton D. Fuller, MD, PhD (cdfuller@mdanderson.org)

Table S1. Computed tomography- derived intensity histogram, shape and texture analysis features set

Feature Category	Features	Definition	References
Gray Level Cooccurrence Matrix 25<br><br>Gray Level Cooccurrence Matrix 3	Auto-Correlation	The Correlation texture measures the linear dependency of grey levels on those of neighbouring pixels.	1
	Cluster Prominence	A measure of the skewness or asymmetry	1
	Cluster Shade	A measure of the skewness or asymmetry	1
	Cluster Tendency	Assess if non-random structure exists in the data by measuring the probability that the data is generated by a uniform data distribution	1
	Contrast	Returns a measure of the intensity contrast between a pixel and its neighbor over the whole image.	2, 3
	Correlation	Returns a measure of how correlated a pixel is to its neighbor over the whole image.	2, 3
	Difference Entropy		2, 4
	Dissimilarity		1
	Energy		2, 3
	Entropy		1
	Homogeneity		2, 3, 4
	Homogeneity 2		2, 3, 4
	Information Measure Correlation 1		2, 3, 4
	Information Measure Correlation 2		2, 3, 4
	Inverse Diff Moment Norm		2, 3, 4
	Inverse Diff Norm		2, 3, 4

	Inverse Variance		4
	Max Probability		1
	Sum Average		2, 3, 4
	Sum Entropy		2, 3, 4
	Sum Variance		2, 3, 4
	Variance		4
GrayLevelRunLengthMatrix25	Gray Level Non-uniformity		5
	High Gray Level Run Empha		5
	Long Run Emphasis		5
	Long Run High Gray Level Empha		5
	Long Run Low Gray Level Empha		5
	Low Gray Level Run Empha		5
	Run Length Non-uniformity		5
	Run Percentage		5
	Short Run Emphasis		5
	Short Run High Gray Level Empha		5
	Short Run Low Gray Level Empha		5

Neighbor Intensity Difference 25<br><br>Neighbor Intensity Difference 3	Busyness		6
	Coarseness		6
	Complexity		6
	Contrast		6
	Texture Strength		6
Intensity Direct	Energy		4
	Global Entropy	The intensity entropy among all the voxels	4
	Global Max	The intensity maximum among all the voxels.	4
	Global Mean	The intensity mean among all the voxels.	4
	Global Median	The intensity median among all the voxels.	4
	Global Min	The intensity minimum among all the voxels.	4
	Global Std	The intensity standard deviation among all the voxels.	4
	Global Uniformity	The intensity uniformity among all the voxels.	4
	Inter-Quartile Range	The interquartile range of the intensity values among all the voxels.	4
	Kurtosis	Measure the peakedness of all the voxels' intensity.	4
	Local Entropy Max	First, at each voxel, compute entropy in its neighborhood region. Then, compute the maximum among all the voxel's entropy calculated from step 1.	4
	Local Entropy Mean	First, at each voxel, compute entropy in its neighborhood region. Then, compute the mean among all the voxel's entropy calculated from step 1.	4
	Local Entropy Median	First, at each voxel, compute entropy in its neighborhood region. Then, compute the median among all the voxel's entropy calculated from step 1.	4
	Local Entropy Min	First, at each voxel, compute entropy in its neighborhood region. Then, compute the minimum among all the voxel's entropy calculated from step 1.	4
	Local Entropy Std	First, at each voxel, compute entropy in its neighborhood region. Then, compute the standard deviation among all the voxel's entropy calculated from step 1.	4

	Local Range Max	First, at each voxel, compute range value (Max Value-Min Value) in its neighborhood region. Then, compute the median among all the voxel's range value calculated from step 1.	4
	Local Range Mean	First, at each voxel, compute range value (Max Value-Min Value) in its neighborhood region. Then, compute the mean among all the voxel's range value calculated from step 1.	4
	Local Range Median	First, at each voxel, compute range value (Max Value-Min Value) in its neighborhood region. Then, compute the median among all the voxel's range value calculated from step 1.	4
	Local Range Min	First, at each voxel, compute range value (Max Value-Min Value) in its neighborhood region. Then, compute the minimum among all the voxel's range value calculated from step 1.	4
	Local Range Std	First, at each voxel, compute range value (Max Value-Min Value) in its neighborhood region. Then, compute the standard deviation among all the voxel's range value calculated from step 1.	4
	Local Std Max	First, at each voxel, compute standard deviation in its neighborhood region. Then, compute the maximum among all the voxel's standard deviation value calculated from step 1.	4
	Local Std Mean	First, at each voxel, compute standard deviation in its neighborhood region. Then, compute the mean among all the voxel's standard deviation value calculated from step 1.	4
	Local Std Median	First, at each voxel, compute standard deviation in its neighborhood region. Then, compute the median among all the voxel's standard deviation value calculated from step 1.	4
	Local Std Min	First, at each voxel, compute standard deviation in its neighborhood region. Then, compute the minimum among all the voxel's standard deviation value calculated from step 1.	4
	Local Std Std	First, at each voxel, compute standard deviation in its neighborhood region. Then, compute the standard deviation all the voxel's standard deviation value calculated from step 1.	4
	Mean Absolute Deviation	The mean absolute deviation of the intensity values among all the voxels.	4
	Median Absolute Deviation	The median absolute deviation of the intensity values among all the voxels.	4
	Percentile	Percentiles of the intensity values among all the voxels.	4

	Quantile	Quantiles of the intensity values among all the voxels.	4
	Range	The intensity range (Max Value-Min Value) among all the voxels.	4
	Root Mean Square		4
	Skewness	Measure the asymmetry of all the voxels' intensity.	4
	Variance		4
Intensity Histogram	Inter-Quartile Range	The interquartile range of the occurrence probability values in the histogram.	4
	Kurtosis	Measure the peakedness of the occurrence probability values in the histogram.	4
	Mean Absolute Deviation	The mean absolute deviation of the occurrence probability values in the histogram.	4
	Median Absolute Deviation	The median absolute deviation of the occurrence probability values in the histogram.	4
	Percentile	Percentiles of the occurrence probability values in the histogram.	4
	Percentile Area	Percentiles of values in the accumulative histogram.	4
	Quantile	Quantiles of the occurrence probability values in the histogram.	4
	Range	Measures the range (Max Value-Min Value) of the occurrence probability values in the histogram.	4
	Skewness	Measure the asymmetry of the occurrence probability values in the histogram.	4
Shape	Compactness 1	Compactness1= (Volume)/(sqrt(pi)*(SurfaceArea)^(2/3)	4
	Compactness 2	Compactness2= 36*pi*(Volume^2)/((SurfaceArea)^3).	4
	Convex	Measure the proportion of the pixels in the convex hull that are also in the region.	4
	Convex Hull Volume	The mean volume of the 2D convex hulls that are the convex envelopes of each slice's binary mask.	4
	Convex Hull Volume 3D	3D volume of the convex hull that is the convex envelope of binary mask.	4
	Mass		4
	Max 3D Diameter	Max 3D Diameter= largest pairwise Euclidean distance between voxels on the surface of the tumor volume.	4

	Mean Breadth	Denotes integral of mean curvature	4
	Number Of Voxel	The number of voxels treating the edge voxels differently.	4
	Orientation	Measures the angle between the x-axis and the major axis of the ellipse in 2D.	4
	Roundness	Measures how much the binary mask is close to circle in 2D.	4
	Spherical Disproportion		4
	Sphericity		4
	Surface Area	The surface area of the binary mask.	7
	Surface Area Density	Surface Area Density= (surface area of the binary mask)/(volume of the binary mask).	4, 7
	Volume	The physical volume treating the edge voxels differently.	7

References

1 Soh, L. K. & Tsatsoulis, C. Texture analysis of SAR sea ice imagery using gray level co-occurrence matrices. *IEEE Transactions on Geoscience and Remote Sensing* **37**, 780-795, doi:10.1109/36.752194 (1999).

2 Haralick, R. M., Shanmugam, K. & Dinstein, I. Textural Features for Image Classification. *IEEE Transactions on Systems, Man, and Cybernetics* **SMC-3**, 610-621, doi:10.1109/TSMC.1973.4309314 (1973).

3 Haralick, R. M. & Shapiro, L. G. *Computer and Robot Vision*. (Addison-Wesley Longman Publishing Co., Inc., 1992).

4 Aerts, H. J. W. L. *et al.* Decoding tumour phenotype by noninvasive imaging using a quantitative radiomics approach. *Nature Communications* **5**, 4006, doi:10.1038/ncomms5006

http://www.nature.com/articles/ncomms5006#supplementary-information (2014).

5 Xiaoou, T. Texture information in run-length matrices. *IEEE Transactions on Image Processing* **7**, 1602-1609, doi:10.1109/83.725367 (1998).

6 Amadasun, M. & King, R. Texural Features Corresponding to Texural Properties. *IEEE Transactions on Systems, Man and Cybernetics* **19**, 1264-1274, doi:10.1109/21.44046 (1989).

7 Legland, D., Kiêu, K. & Devaux, M.-F. COMPUTATION OF MINKOWSKI MEASURES ON 2D AND 3D BINARY IMAGES. *2011* **26**, 10, doi:10.5566/ias.v26.p83-92 (2011).
